# ACTIVITIES OF DAILY LIVING (ADL/IADL) ASSISTANCE AND DEPRESSION AMONG COMMUNITY-DWELLING OLDER ADULTS IN THE US

**DOI:** 10.1093/geroni/igae098.0233

**Published:** 2024-12-31

**Authors:** Debasree Das Gupta, Uma Kelekar

**Affiliations:** Utah State University, Logan, Utah, United States; Marymount University, Arlington, Virginia, United States

## Abstract

Limitations in daily and instrumental activities of daily living (ADL/IADL) are among the strongest predictors of late-life depression, while ADL/IADL assistance has the potential to reduce depression among older adults. Prior research in this area has predominantly focused on proximate (e.g., going without food when hungry, wetting/soiling bed or clothes) or healthcare (e.g., hospitalizations, nursing home placements) outcomes of unmet ADL/IADL needs. Thus, the risk/protective association of ADL/IADL assistance with late-life depression remains unclear. To address this gap, we evaluated the longitudinal association between ADL/IADL assistance and subsequent depression using data from a nationally representative sample of community-dwelling Americans age 65 and older (n=5,176) who participated in the 2016-2018 Health and Retirement Study. We conducted bivariate and multivariable (logistic regression) analyses to estimate: 1) the proportion of older adults reporting ADL/IADL difficulties but not getting ADL/IADL help, and 2) the odds of subsequent depression among those reporting no versus one or more help with ADL/IADL (reference: those reporting no ADL/IADL difficulties). Results indicated a higher proportion of older adults did not get help with ADL (6.4%) compared to no help with IADL (1.9%). However, the adjusted odds of subsequent depression associated with no IADL assistance (adjusted odds ratio [aOR]: 3.22; 95% confidence interval [CI]: 2.19,4.78) was 1.66 times higher than that associated with no ADL assistance (aOR: 1.94; 95% CI: 1.35,2.78). Given these findings, enhancing long-term care services for older adults would be essential for meeting the current national goal of reducing mental distress among persons living with disabilities.

